# Supplementary material for: The role of microglia and their CX3CR1 signaling in adult neurogenesis in the olfactory bulb
Source: eLife. 2017 Dec 18;6:e30809. doi: 10.7554/eLife.30809 (PMC5734876; doi:10.7554/eLife.30809)
Supplement: Supplementary file 1. [file elife-30809-supp1.docx]

|  | | | | | | | | | |
| --- | --- | --- | --- | --- | --- | --- | --- | --- | --- |
| Supplementary file 1: Gene transcripts significantly differentially regulated in the olfactory bulb of PLX5622-treated mice, compared with control diet-treated mice. Down regulated genes are marked in blue; Up regulated genes are marked in pink. *n*=3 mice in each group. | | | | | | | | | |
| Gene | **Entrez Gene Name** | **Log 2 Fold Change** | | | | **p-value** | **Location** | **Type** | |
|  |  |  |  |  |  |  |  |  |  |
| Microglia enriched genes | | | | | | | | | |
| *Mrc1* | mannose receptor, C type 1 | | | -4.482 | 5.25E-22 | | Plasma Membrane | | transmembrane receptor |
| *Pf4* | platelet factor 4 | | | -3.324 | 1.76E-04 | | Extracellular Space | | cytokine |
| *F13a1* | coagulation factor XIII, A1 polypeptide | | | -3.163 | 1.71E-07 | | Extracellular Space | | enzyme |
| *Fcrls* | Fc receptor-like S, scavenger receptor | | | -2.945 | 1.45E-31 | | Plasma Membrane | | other |
| *Ltc4s* | leukotriene C4 synthase | | | -2.682 | 3.14E-09 | | Cytoplasm | | enzyme |
| *Cd86* | CD86 molecule | | | -2.581 | 2.99E-10 | | Plasma Membrane | | transmembrane receptor |
| *Ms4a7* | membrane-spanning 4-domains, subfamily A, member 7 | | | -2.531 | 3.91E-03 | | Other | | other |
| *Crybb1* | crystallin, beta B1 | | | -2.424 | 7.79E-09 | | Other | | other |
| *P2ry13* | purinergic receptor P2Y, G-protein coupled, 13 | | | -2.377 | 1.53E-12 | | Plasma Membrane | | G-protein coupled receptor |
| *Susd3* | sushi domain containing 3 | | | -2.303 | 3.97E-04 | | Plasma Membrane | | other |
| *Cysltr1* | cysteinyl leukotriene receptor 1 | | | -2.292 | 1.15E-03 | | Plasma Membrane | | G-protein coupled receptor |
| *Slc2a5* | solute carrier family 2 (facilitated glucose/fructose transporter), member 5 | | | -2.216 | 1.45E-06 | | Plasma Membrane | | transporter |
| *Abcc3* | ATP-binding cassette, sub-family C (CFTR/MRP), member 3 | | | -2.213 | 1.74E-09 | | Plasma Membrane | | transporter |
| *Clec5a* | C-type lectin domain family 5, member A | | | -2.186 | 2.50E-05 | | Plasma Membrane | | other |
| *P2ry12* | purinergic receptor P2Y, G-protein coupled, 12 | | | -2.181 | 2.99E-17 | | Plasma Membrane | | G-protein coupled receptor |
| *Lag3* | lymphocyte-activation gene 3 | | | -2.123 | 1.69E-26 | | Plasma Membrane | | transmembrane receptor |
| *Siglecf* | sialic acid binding Ig-like lectin 8 | | | -2.123 | 3.80E-03 | | Plasma Membrane | | transmembrane receptor |
| *C5ar2* | complement component 5a receptor 2 | | | -2.077 | 5.86E-03 | | Plasma Membrane | | G-protein coupled receptor |
| *Adgre1* | adhesion G protein-coupled receptor E1 | | | -2.076 | 2.32E-18 | | Plasma Membrane | | G-protein coupled receptor |
| *Siglech* | sialic acid binding Ig-like lectin H | | | -2.022 | 8.55E-11 | | Plasma Membrane | | other |
| *Stab1* | stabilin 1 | | | -1.987 | 1.20E-27 | | Plasma Membrane | | transporter |
| *Trem2* | triggering receptor expressed on myeloid cells 2 | | | -1.984 | 2.50E-23 | | Plasma Membrane | | transmembrane receptor |
| *Nlrp1b* | NLR family, pyrin domain containing 1 | | | -1.916 | 3.52E-03 | | Cytoplasm | | other |
| *Ebi3* | Epstein-Barr virus induced 3 | | | -1.893 | 3.86E-03 | | Extracellular Space | | cytokine |
| *Cx3cr1* | chemokine (C-X3-C motif) receptor 1 | | | -1.822 | 3.59E-15 | | Plasma Membrane | | G-protein coupled receptor |
| *Tlr1* | toll-like receptor 1 | | | -1.792 | 4.81E-03 | | Plasma Membrane | | transmembrane receptor |
| *Ang* | angiogenin, ribonuclease, RNase A family, 5 | | | -1.777 | 7.33E-03 | | Extracellular Space | | enzyme |
| *Csf1r* | colony stimulating factor 1 receptor | | | -1.746 | 6.08E-14 | | Plasma Membrane | | kinase |
| *Lst1* | leukocyte specific transcript 1 | | | -1.736 | 5.18E-03 | | Cytoplasm | | other |
| *Pik3cg* | phosphatidylinositol-4,5-bisphosphate 3-kinase, catalytic subunit gamma | | | -1.726 | 3.37E-07 | | Cytoplasm | | kinase |
| *Il16* | interleukin 16 | | | -1.709 | 8.41E-06 | | Extracellular Space | | cytokine |
| *C1qa* | complement component 1, q subcomponent, A chain | | | -1.658 | 1.01E-05 | | Extracellular Space | | other |
| *Cd37* | CD37 molecule | | | -1.621 | 1.95E-05 | | Plasma Membrane | | other |
| *Hpgds* | hematopoietic prostaglandin D synthase | | | -1.614 | 9.02E-09 | | Cytoplasm | | enzyme |
| *C1qb* | complement component 1, q subcomponent, B chain | | | -1.597 | 4.66E-05 | | Extracellular Space | | other |
| *Gpr34* | G protein-coupled receptor 34 | | | -1.591 | 4.55E-05 | | Plasma Membrane | | G-protein coupled receptor |
| *Fcgr3* | Fc fragment of IgG, low affinity IIa, receptor (CD32) | | | -1.589 | 6.49E-07 | | Plasma Membrane | | transmembrane receptor |
| *Csf3r* | colony stimulating factor 3 receptor (granulocyte) | | | -1.582 | 2.31E-07 | | Plasma Membrane | | transmembrane receptor |
| *Cd163* | CD163 molecule | | | -1.566 | 6.31E-03 | | Plasma Membrane | | transmembrane receptor |
| *Itgam* | integrin, alpha M (complement component 3 receptor 3 subunit) | | | -1.549 | 7.86E-05 | | Plasma Membrane | | transmembrane receptor |
| *C1qc* | complement component 1, q subcomponent, C chain | | | -1.544 | 3.41E-06 | | Extracellular Space | | other |
| *Tyrobp* | TYRO protein tyrosine kinase binding protein | | | -1.526 | 2.68E-10 | | Plasma Membrane | | transmembrane receptor |
| *AF251705* | CD300c molecule | | | -1.519 | 2.68E-05 | | Plasma Membrane | | transmembrane receptor |
| *Lyz1* | lysozyme | | | -1.501 | 8.71E-03 | | Extracellular Space | | enzyme |
| *Havcr2* | hepatitis A virus cellular receptor 2 | | | -1.555 | 1.08E-03 | | Plasma Membrane | | other |
| *Ecscr* | endothelial cell surface expressed chemotaxis and apoptosis regulator | | | -1.472 | 2.34E-03 | | Other | | other |
| *Tnfaip8l2* | tumor necrosis factor, alpha-induced protein 8-like 2 | | | -1.468 | 2.46E-03 | | Cytoplasm | | other |
| *Ptpn18* | protein tyrosine phosphatase, non-receptor type 18 (brain-derived) | | | -1.464 | 3.52E-03 | | Nucleus | | phosphatase |
| *Adrb2* | adrenoceptor beta 2, surface | | | -1.463 | 8.66E-03 | | Plasma Membrane | | G-protein coupled receptor |
| *Nckap1l* | NCK-associated protein 1-like | | | -1.455 | 5.23E-09 | | Plasma Membrane | | other |
| *Cd53* | CD53 molecule | | | -1.452 | 2.55E-06 | | Plasma Membrane | | other |
| *Selplg* | selectin P ligand | | | -1.445 | 2.75E-10 | | Plasma Membrane | | other |
| *Ctss* | cathepsin S | | | -1.398 | 1.33E-04 | | Cytoplasm | | peptidase |
| *Tlr7* | toll-like receptor 7 | | | -1.382 | 3.66E-03 | | Plasma Membrane | | transmembrane receptor |
| *Ccr5* | chemokine (C-C motif) receptor 5 (gene/pseudogene) | | | -1.378 | 7.97E-03 | | Plasma Membrane | | G-protein coupled receptor |
| *Laptm5* | lysosomal protein transmembrane 5 | | | -1.331 | 1.20E-06 | | Plasma Membrane | | other |
| *Hexb* | hexosaminidase B (beta polypeptide) | | | -1.268 | 3.15E-16 | | Cytoplasm | | enzyme |
| *Mlxipl* | MLX interacting protein-like | | | -1.249 | 6.31E-05 | | Nucleus | | transcription regulator |
| *Cd33* | CD33 antigen | | | -1.246 | 2.39E-03 | | Plasma Membrane | | other |
| *Pik3r5* | phosphoinositide-3-kinase, regulatory subunit 5 | | | -1.243 | 9.21E-04 | | Cytoplasm | | kinase |
| *Rnase4* | ribonuclease, RNase A family, 4 | | | -1.236 | 2.98E-11 | | Extracellular Space | | enzyme |
| *Inpp5d* | inositol polyphosphate-5-phosphatase D | | | -1.227 | 2.71E-07 | | Cytoplasm | | phosphatase |
| *Tmem119* | transmembrane protein 119 | | | -1.224 | 8.16E-11 | | Cytoplasm | | other |
| *Aif1* | allograft inflammatory factor 1 | | | -1.213 | 1.54E-03 | | Nucleus | | other |
| *Sash3* | SAM and SH3 domain containing 3 | | | -1.211 | 1.98E-03 | | Cytoplasm | | other |
| *Wdfy4* | WDFY family member 4 | | | -1.206 | 1.29E-03 | | Other | | other |
| *Mlph* | melanophilin | | | -1.192 | 1.39E-03 | | Cytoplasm | | other |
| *Tlr13* | toll-like receptor 13 | | | -1.187 | 8.31E-03 | | Cytoplasm | | other |
| *Fgd2* | FYVE, RhoGEF and PH domain containing 2 | | | -1.169 | 1.40E-07 | | Cytoplasm | | other |
| *Tlr9* | toll-like receptor 9 | | | -1.154 | 3.38E-03 | | Plasma Membrane | | transmembrane receptor |
| *Cd68* | CD68 molecule | | | -1.131 | 3.79E-05 | | Plasma Membrane | | other |
| *Ncf1* | neutrophil cytosolic factor 1 | | | -1.133 | 6.47E-04 | | Cytoplasm | | enzyme |
| *Lyl1* | lymphoblastic leukemia associated hematopoiesis regulator 1 | | | -1.127 | 1.73E-03 | | Nucleus | | transcription regulator |
| *Alox5ap* | arachidonate 5-lipoxygenase-activating protein | | | -1.122 | 5.68E-03 | | Plasma Membrane | | other |
| *Il10ra* | interleukin 10 receptor, alpha | | | -1.096 | 2.52E-04 | | Plasma Membrane | | transmembrane receptor |
| *Hcls1* | hematopoietic cell-specific Lyn substrate 1 | | | -1.072 | 4.69E-03 | | Nucleus | | other |
| *Parvg* | parvin, gamma | | | -1.066 | 2.45E-03 | | Cytoplasm | | other |
| *Pld4* | phospholipase D family, member 4 | | | -1.052 | 6.55E-06 | | Extracellular Space | | enzyme |
| *Vav1* | vav 1 guanine nucleotide exchange factor | | | -1.048 | 6.88E-03 | | Nucleus | | transcription regulator |
| *Tbxas1* | thromboxane A synthase 1 (platelet) | | | -1.041 | 7.05E-03 | | Plasma Membrane | | enzyme |
| *Blnk* | B-cell linker | | | -1.012 | 4.03E-03 | | Cytoplasm | | other |
| *Abca9* | ATP-binding cassette, sub-family A (ABC1), member 9 | | | -1.003 | 5.24E-07 | | Cytoplasm | | transporter |
| *Mpeg1* | macrophage expressed 1 | | | -0.954 | 2.65E-07 | | Cytoplasm | | other |
| *Ncf2* | neutrophil cytosolic factor 2 | | | -0.947 | 9.47E-03 | | Cytoplasm | | enzyme |
| *Unc93b1* | unc-93 homolog B1 (C. elegans) | | | -0.94 | 2.02E-08 | | Cytoplasm | | transporter |
| *Fermt3* | fermitin family member 3 | | | -0.938 | 4.29E-04 | | Cytoplasm | | enzyme |
| *Cyth4* | cytohesin 4 | | | -0.925 | 9.00E-06 | | Cytoplasm | | other |
| *Hmha1* | histocompatibility (minor) HA-1 | | | -0.913 | 3.30E-04 | | Cytoplasm | | transporter |
| *Dock2* | dedicator of cytokinesis 2 | | | -0.889 | 9.32E-04 | | Cytoplasm | | other |
| *Slco2b1* | solute carrier organic anion transporter family, member 2B1 | | | -0.882 | 3.38E-07 | | Plasma Membrane | | transporter |
| *Dock8* | dedicator of cytokinesis 8 | | | -0.852 | 3.77E-03 | | Cytoplasm | | other |
| *Tnfrsf1b* | tumor necrosis factor receptor superfamily, member 1B | | | -0.845 | 6.70E-03 | | Plasma Membrane | | transmembrane receptor |
| *Lpcat2* | lysophosphatidylcholine acyltransferase 2 | | | -0.835 | 1.94E-05 | | Cytoplasm | | enzyme |
| *Arhgap30* | Rho GTPase activating protein 30 | | | -0.819 | 9.38E-03 | | Cytoplasm | | other |
| *Abi3* | ABI family, member 3 | | | -0.799 | 4.19E-05 | | Plasma Membrane | | other |
| *Adcy7* | adenylate cyclase 7 | | | -0.781 | 1.06E-04 | | Plasma Membrane | | enzyme |
| *Ptpn6* | protein tyrosine phosphatase, non-receptor type 6 | | | -0.781 | 7.41E-03 | | Cytoplasm | | phosphatase |
| *Cd84* | CD84 molecule | | | -0.767 | 1.05E-03 | | Plasma Membrane | | other |
| *F11r* | F11 receptor | | | -0.766 | 2.61E-03 | | Plasma Membrane | | other |
| *Rab3il1* | RAB3A interacting protein (rabin3)-like 1 | | | -0.764 | 7.94E-04 | | Other | | other |
| *Mafb* | v-maf avian musculoaponeurotic fibrosarcoma oncogene homolog B | | | -0.744 | 5.63E-04 | | Nucleus | | transcription regulator |
| *Lair1* | leukocyte-associated immunoglobulin-like receptor 1 | | | -0.721 | 2.51E-04 | | Plasma Membrane | | transmembrane receptor |
| *Ptgs1* | prostaglandin-endoperoxide synthase 1 (prostaglandin G/H synthase and cyclooxygenase) | | | -0.714 | 8.40E-04 | | Cytoplasm | | enzyme |
| *Olfml3* | olfactomedin-like 3 | | | -0.653 | 4.51E-03 | | Extracellular Space | | other |
| *Tgfbr1* | transforming growth factor, beta receptor 1 | | | -0.624 | 3.53E-05 | | Plasma Membrane | | kinase |
| *Maf* | v-maf avian musculoaponeurotic fibrosarcoma oncogene homolog | | | -0.566 | 1.09E-03 | | Nucleus | | transcription regulator |
| *Cebpa* | CCAAT/enhancer binding protein (C/EBP), alpha | | | -0.551 | 2.16E-03 | | Nucleus | | transcription regulator |
| *Itgb5* | integrin, beta 5 | | | -0.522 | 8.71E-06 | | Plasma Membrane | | other |
| *Map3k14* | mitogen-activated protein kinase kinase kinase 14 | | | -0.517 | 2.10E-03 | | Cytoplasm | | kinase |
| *Entpd1* | ectonucleoside triphosphate diphosphohydrolase 1 | | | -0.514 | 1.96E-03 | | Plasma Membrane | | enzyme |
| *Rgs10* | regulator of G-protein signaling 10 | | | -0.489 | 1.10E-06 | | Cytoplasm | | other |
| *P2rx7* | purinergic receptor P2X, ligand gated ion channel, 7 | | | -0.444 | 4.40E-03 | | Plasma Membrane | | ion channel |
| *Other genes* | | | | | | | | | |
| *Bank1* | B-cell scaffold protein with ankyrin repeats 1 | | -3.377 | | 4.42E-03 | | Extracellular Space | | other |
| *Klk8* | kallikrein-related peptidase 8 | | -1.666 | | 9.76E-03 | | Extracellular Space | | peptidase |
| *Gna15* | guanine nucleotide binding protein (G protein), alpha 15 (Gq class) | | -1.638 | | 4.43E-04 | | Plasma Membrane | | enzyme |
| *4632428N05Rik* | chromosome 10 open reading frame 54 | | -1.534 | | 1.14E-18 | | Plasma Membrane | | other |
| *Tifab* | TRAF-interacting protein with forkhead-associated domain, family member B | | -1.514 | | 5.42E-05 | | Other | | other |
| *Itpripl1* | inositol 1,4,5-trisphosphate receptor interacting protein-like 1 | | -1.371 | | 1.10E-03 | | Other | | other |
| *Fyb* | FYN binding protein | | -1.027 | | 5.29E-04 | | Nucleus | | other |
| *Gal3st4* | galactose-3-O-sulfotransferase 4 | | -0.891 | | 1.81E-04 | | Cytoplasm | | enzyme |
| *Fes* | FES proto-oncogene, tyrosine kinase | | -0.855 | | 3.33E-03 | | Cytoplasm | | kinase |
| *Fam105a* | family with sequence similarity 105, member A | | -0.687 | | 2.65E-05 | | Other | | other |
| *Mob3c* | MOB kinase activator 3C | | -0.619 | | 6.39E-03 | | Other | | other |
| *Ctsh* | cathepsin H | | -0.609 | | 2.46E-03 | | Cytoplasm | | peptidase |
| *Golm1* | golgi membrane protein 1 | | -0.595 | | 8.28E-04 | | Cytoplasm | | other |
| *Il10rb* | interleukin 10 receptor, beta | | -0.532 | | 3.11E-03 | | Plasma Membrane | | transmembrane receptor |
| *Rps6ka1* | ribosomal protein S6 kinase, 90kDa, polypeptide 1 | | -0.478 | | 8.47E-03 | | Cytoplasm | | kinase |
| *Ifi27l2a* | interferon, alpha-inducible protein 27 like 2A | | 0.643 | | 6.19E-03 | | Other | | other |
| *Gm7120* | chromosome 5 open reading frame 28 | | 0.825 | | 1.54E-03 | | Other | | other |
| *Clec3b* | C-type lectin domain family 3, member B | | 0.839 | | 3.01E-03 | | Extracellular Space | | other |
| *Ccnb1* | cyclin B1 | | 3.125 | | 1.26E-03 | | Cytoplasm | | kinase |
