# Supplementary material for: The role of microglia and their CX3CR1 signaling in adult neurogenesis in the olfactory bulb
Source: eLife. 2017 Dec 18;6:e30809. doi: 10.7554/eLife.30809 (PMC5734876; doi:10.7554/eLife.30809)
Supplement: Supplementary file 2. [file elife-30809-supp2.docx]

| **Supplementary file 2:** Comparisons between gene transcript expression of 40 cytokines and chemokines in the olfactory bulbs of *Cx3cr1^-/-^* (KO) and PLX5622-treated (PLX) mice, as well as their respective wild type (WT) and control diet-treated (CON) mice. NE= No expression. *n*= 3 mice in each group. | | | | | | | |
| --- | --- | --- | --- | --- | --- | --- | --- |
| **Gene annotation** |  | **KO VS. WT** | | **PLX VS. CON** | | **KO VS. PLX** | |
|  |  | Log 2 Fold Change | q value | Log 2 Fold Change | q value | Log 2 Fold Change | q value |
| *Il1α* |  | 1.004 | 0.856 | -0.996 | 0.999 | 1.452 | 0.749 |
| *Il1β* |  | -1.58 | 0.694 | -0.33 | 0.999 | -2.114 | 0.999 |
| *Il2* |  | NE | NE | NE | NE | NE | NE |
| *Il3* |  | 0.034 | 0.999 | -3.259 | 0.999 | -0.015 | 0.999 |
| *Il4* |  | -0.194 | 0.999 | 0.871 | 0.999 | -0.244 | 0.999 |
| *Il5* |  | -0.959 | 0.949 | -1.928 | 0.999 | 0.192 | 0.999 |
| *Il6* |  | 3.359 | 0.9 | -3.154 | 0.999 | 6.481 | 0.87 |
| *Il7* |  | -0.54 | 0.999 | 0.423 | 0.999 | -1.009 | 0.999 |
| *Il10* |  | -3.124 | 0.949 | -4.778 | 0.999 | -0.015 | 0.999 |
| *Il12a* |  | -1.231 | 0.821 | 0.321 | 0.999 | -1.508 | 0.828 |
| *Il13* |  | 1.014 | 0.999 | 1.065 | 0.999 | -0.085 | 0.999 |
| *Il15* |  | -0.119 | 0.999 | -0.094 | 0.999 | 0.11 | 0.999 |
| *Il16* |  | -0.215 | 0.949 | -1.709 | 0.004 | 1.477 | 0.03 |
| *Il17α* |  | NE | NE | NE | NE | NE | NE |
| *Il23α* |  | 0.316 | 0.999 | 0.706 | 0.999 | -0.408 | 0.999 |
| *Il27* |  | 1.04 | 0.951 | -0.958 | 0.999 | 0.964 | 0.999 |
| *Il1ra* |  | -1.212 | 0.9 | -1.341 | 0.999 | 1 | 0.691 |
| Ifng |  | -0.965 | 0.999 | -0.017 | 0.999 | -0.063 | 0.999 |
| Tnfα |  | 1.679 | 0.949 | -1.475 | 0.999 | 1.85 | 0.999 |
| *Gcsf* |  | 2.49 | 0.865 | -1.936 | 0.999 | 4.405 | 0.768 |
| *Gmcsf* |  | -0.012 | 0.999 | 4.189 | 0.999 | 0.56 | 0.999 |
| *Mcsf* |  | 0.084 | 0.949 | 0.014 | 0.999 | -0.048 | 0.999 |
| *Trem1* |  | 2.343 | 0.949 | 0.987 | 0.999 | 1.236 | 0.999 |
| *Cxcl13* |  | 2.484 | 0.898 | 1 | 0.999 | -1.072 | 0.999 |
| *Ccl1* |  | NE | NE | NE | NE | NE | NE |
| *Ccl11* |  | 2.021 | 0.857 | -0.532 | 0.999 | 1.555 | 0.999 |
| *C5a* |  | 0.214 | 0.999 | -0.136 | 0.999 | -0.041 | 0.999 |
| *Cxcl1* |  | 0.263 | 0.638 | 1.464 | 0.999 | -0.585 | 0.668 |
| *Ccl2* |  | 0.656 | 0.994 | 0.768 | 0.999 | 1.106 | 0.999 |
| *Ccl12* |  | 2.183 | 0.84 | 0.022 | 0.999 | 1.663 | 0.999 |
| *Cxcl9* |  | 0.315 | 0.997 | -0.826 | 0.999 | 1.061 | 0.999 |
| *Ccl3* |  | 0.819 | 0.944 | 0.398 | 0.999 | 0.596 | 0.999 |
| *Ccl4* |  | 0.561 | 0.974 | -0.492 | 0.999 | 1.039 | 0.999 |
| *Cxcl2* |  | 2.932 | 0.949 | -0.042 | 0.999 | 4.889 | 0.999 |
| *Ccl5* |  | -1.322 | 0.627 | 0.87 | 0.999 | -2.304 | 0.021 |
| *Cxcl12* |  | 0.271 | 0.87 | 0.139 | 0.999 | 0.342 | 0.925 |
| *Ccl17* |  | -1.618 | 0.76 | 0.464 | 0.999 | -0.455 | 0.999 |
| *Timp1* |  | 0.754 | 0.644 | 0.116 | 0.999 | -0.562 | 0.999 |
| *Cxcl11* |  | -1.97 | 0.949 | 1.61 | 0.999 | -2.606 | 0.999 |
| *Icam1* |  | 0.504 | 0.949 | -0.073 | 0.999 | 0.628 | 0.999 |
